# Cholesterol modulates presynaptic and postsynaptic properties of excitatory synaptic transmission

**DOI:** 10.1038/s41598-020-69454-5

**Published:** 2020-07-28

**Authors:** Miloslav Korinek, Inmaculada M. Gonzalez-Gonzalez, Tereza Smejkalova, Dragana Hajdukovic, Kristyna Skrenkova, Jan Krusek, Martin Horak, Ladislav Vyklicky

**Affiliations:** 1 Department of Cellular Neurophysiology, Institute of Physiology of the Czech Academy of Sciences, Videnska 1083, Prague, Czech Republic; 2 School of Pharmacy and Biomedical Sciences, University of Central Lancashire, Preston, UK

**Keywords:** Lipid-storage diseases, Neurodegeneration, Ion channels in the nervous system, Synaptic vesicle exocytosis, Neurophysiology

## Abstract

Cholesterol is a structural component of cellular membranes particularly enriched in synapses but its role in synaptic transmission remains poorly understood. We used rat hippocampal cultures and their acute cholesterol depletion by methyl-β-cyclodextrin as a tool to describe the physiological role of cholesterol in glutamatergic synaptic transmission. Cholesterol proved to be a key molecule for the function of synapses as its depletion resulted in a significant reduction of both NMDA receptor (NMDAR) and AMPA/kainate receptor-mediated evoked excitatory postsynaptic currents (eEPSCs), by 94% and 72%, respectively. We identified two presynaptic and two postsynaptic steps of synaptic transmission which are modulated by cholesterol and explain together the above-mentioned reduction of eEPSCs. In the postsynapse, we show that physiological levels of cholesterol are important for maintaining the normal probability of opening of NMDARs and for keeping NMDARs localized in synapses. In the presynapse, our results favour the hypothesis of a role of cholesterol in the propagation of axonal action potentials. Finally, cholesterol is a negative modulator of spontaneous presynaptic glutamate release. Our study identifies cholesterol as an important endogenous regulator of synaptic transmission and provides insight into molecular mechanisms underlying the neurological manifestation of diseases associated with impaired cholesterol synthesis or decomposition.

## Introduction

The majority of excitatory neurotransmission in the brain relies on glutamatergic synapses. All steps of neurotransmission, including action potential conduction, neurotransmitter release, and postsynaptic receptor activation occur in the context of plasma membrane and are fundamentally dependent on plasma membrane function. Therefore, in addition to detailed characterization of various synaptic proteins, it is critical to study plasma membrane lipids as potential modulators of synaptic transmission^1–4^.

Cholesterol is a major component of membranes in mammalian cells. Recently, we found that acute cholesterol depletion from neuronal membranes by methyl-β-cyclodextrin (MβCD) induced a substantial decrease of the probability of opening of NMDA receptors (NMDAR) while AMPA/kainate receptor (AMPAR) function was unaffected^3^. This showed that naturally occurring cholesterol controls the function of NMDARs. Plasma membrane cholesterol distribution is not uniform and both presynaptic and postsynaptic membranes are reported to be enriched in cholesterol^5,6^ which suggests an important role of cholesterol in synaptic transmission.

Several studies focused on the effect of cholesterol on long-term potentiation with ambiguous results. Acute cholesterol depletion by cyclodextrins impaired long-term potentiation^7–9^. On the contrary, cholesterol depletion using cholesterol oxidase, which catalyses cholesterol degradation to cholest-4-en-3-one, induced a significant enhancement of long-term potentiation^10^.

At the system level, the concentration of cholesterol in the brain is high compared to other tissues^11^ and it varies under different physiological and pathophysiological conditions. It triples during foetal development and in childhood and it decreases slightly with aging^12,13^. Moreover, some statins (drugs whose pharmacological action is based on the inhibition of endogenous cholesterol biosynthesis), can cross the blood–brain barrier and reduce cholesterol levels in the brain^14^. Niemann-Pick type C disease and Smith-Lemli-Opitz syndrome, both presenting cognitive impairments, are caused by alteration of cholesterol levels^15,16^. Interestingly, intrathecal administration of cyclodextrin is a therapy for Niemann-Pick type C disease currently in clinical trials^17^.

To understand the role of cholesterol in complex processes such as long-term potentiation, effects of cholesterol on synaptic transmission have to be described first. In this study, we performed a comprehensive analysis of the role of naturally occurring cholesterol in excitatory synaptic transmission. Using electrophysiology, immunocytochemistry and single-particle tracking in hippocampal excitatory neurons, we found that cholesterol levels modulate both the presynaptic and the postsynaptic side of synaptic transmission. We show that the role of naturally occurring cholesterol in the postsynapse is (1) to maintain physiological values of the open probability of synaptic NMDARs and (2) to maintain NMDARs within synapses. On the presynaptic side, our experiments are consistent with (3) a hypothesis of an essential role for cholesterol in the propagation of action potentials along axons, and (4) we show the attenuation of spontaneous vesicle release by endogenous cholesterol.

## Results

### Cholesterol modulates both NMDAR and AMPAR eEPSCs

**Figure 1:**
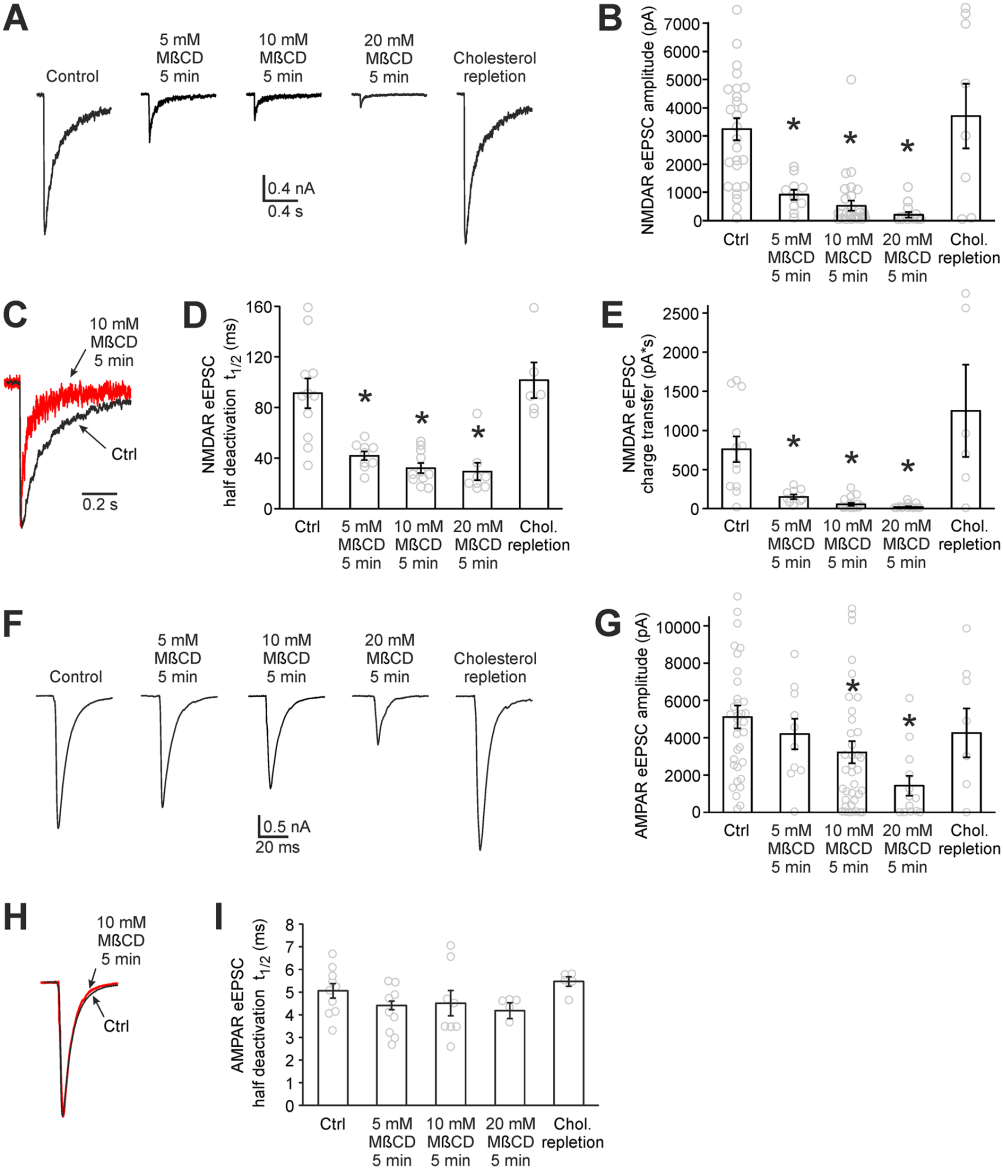
Cholesterol depletion reduces both NMDAR and AMPAR eEPSCs. (**A**) Representative NMDAR eEPSCs measured in hippocampal autaptic neurons under control conditions, after MβCD pretreatment (cholesterol depletion) and after cholesterol repletion. Cholesterol-repleted neurons were pretreated by 20 mM MβCD (5 min) and subsequently by cholesterol/MβCD complex (3.4/20 mM, 10 min). Traces were recorded in the presence of 10 µM CNQX and 10 µM glycine in Mg-free ECS. Action potential signals were removed for clarity. (**B**) Average amplitudes of NMDAR eEPSCs recorded under the conditions described in A. n = 8 to 30 neurons for each bar. (**C**) The comparison of NMDAR eEPSC deactivation of control and 5 min 10 mM MβCD-pretreated neurons. The traces were normalized with respect to their amplitudes and superimposed. (**D**) The effect of cholesterol depletion and cholesterol repletion on NMDAR eEPSC deactivation. Average time of half deactivation (t_1/2_) is displayed. n = 6 to 11 neurons for each bar. (**E**) The effect of cholesterol depletion and repletion on charge transferred by NMDARs in the course of NMDAR eEPSC. n = 7 to 16 neurons for each bar. (**F**) Representative AMPAR eEPSCs recorded under control conditions, after cholesterol depletion and after cholesterol repletion in the presence of 50 µM AP-5 in Mg-free ECS. Action potential signals were removed for clarity. (**G**) Average amplitudes of AMPAR eEPSCs recorded under the same conditions as in F. n = 8 to 37 neurons for each bar. (**H**) The comparison of AMPAR eEPSC time courses of control and 5 min 10 mM MβCD-pretreated neurons. The traces were normalized and superimposed. (**I**) Average time of half deactivation of AMPAR eEPSCs. Cholesterol-depleted and cholesterol-repleted neurons do not differ significantly from controls. n = 5 to 10 neurons for each bar. (*p < 0.05 relative to control, MW test).

Figure 1 shows the effect of cholesterol depletion on NMDAR and AMPAR component of synaptic transmission. NMDAR and AMPAR eEPSCs were recorded in hippocampal autaptic neurons under control conditions and after 5 min of 5, 10 or 20 mM MβCD pretreatment (Fig. 1A–I). MβCD binds plasma membrane cholesterol^18,19^, makes it soluble in water and thus reduces cholesterol level in cultured neurons^3^. Control cells were pretreated in 5, 10 or 20 mM sucrose to experience the same osmotic changes as MβCD-treated samples. Pretreatment in various sucrose concentrations did not induce any significant changes in eEPSCs (NMDAR eEPSC amplitudes were 2780 ± 700 pA, 3110 ± 680 pA and 3920 ± 520 pA for 5, 10 and 20 mM sucrose pretreatment, respectively (*p* = 0.39 ANOVA). AMPAR eEPSC amplitudes were 3820 ± 700 pA, 5260 ± 580 pA and 5520 ± 1040 pA for 5, 10 and 20 mM sucrose pretreatment, respectively (*p* = 0.34 ANOVA)). Therefore, control cell data from various sucrose concentrations were pooled.

Plasma membrane cholesterol depletion resulted in significant changes in both NMDAR and AMPAR eEPSCs (Fig. 1). NMDAR eEPSCs showed a marked reduction of amplitudes as well as a shortening of deactivation times (Fig. 1A–D). The average amplitude of NMDAR eEPSCs decreased 16-fold, from 3250 ± 390 pA (n = 27) in control cells to 205 ± 95 pA (n = 13) in MβCD-pretreated neurons (20 mM MβCD, 5 min, *p* < 0.001, Mann–Whitney (MW) test). To characterize the shortening of NMDAR eEPSCs, we assessed the time of half deactivation (t_1/2,_ Fig. 1C,D). Cholesterol depletion reduced t_1/2_ from 91 ± 12 ms to 29 ± 7 ms (*p* = 0.002, MW test). Both the decrease of amplitude and the shortening of deactivation were reversible by cholesterol repletion (3.4/20 mM cholesterol/MβCD complex, 10 min) showing their dependence on cholesterol and not on other lipids of the plasma membrane. The shortening of NMDAR eEPSC deactivation is in agreement with previous results which described cholesterol depletion-induced increase in desensitization of NMDARs^3^. The electric charge transferred during synaptic activation is the quantity which is physiologically relevant as it is related to the amount of calcium ions entering postsynapses^20^. Figure 1E shows that the charge transferred during NMDAR eEPSC is reduced more than 30-fold following 5 min of 20 mM MβCD pretreatment.

Compared to NMDAR eEPSCs, MβCD pretreatment induced a smaller effect on AMPAR eEPSCs. Their amplitudes were reduced significantly from 5110 ± 620 pA in control neurons to 1420 ± 530 pA in 20 mM MβCD-pretreated neurons (n = 34 and 13 respectively, *p* < 0.001, MW test, Fig. 1F,G). The AMPAR eEPSCs are not significantly different from controls following cholesterol repletion. As regards the time course of AMPAR eEPSCs, it was not altered significantly by MβCD pretreatment (Fig. 1H,I).

Taken together, our data show that cholesterol depletion reduces amplitudes of NMDAR eEPSCs, shortens their deactivations and diminishes charge transferred during NMDAR eEPSCs. Furthermore, cholesterol depletion reduces AMPAR eEPSC amplitudes but this reduction is smaller compared to that of NMDA eEPSCs. It implies that naturally occurring cholesterol in hippocampal neurons is important for the maintenance of both NMDAR and AMPAR eEPSCs and that changes in cholesterol content can substantially modulate excitatory synaptic transmission.

The reduction of eEPSCs in cholesterol-depleted neurons can result from an impairment of various presynaptic and/or postsynaptic steps of synaptic transmission. Our following experiments were aimed at identifying these cholesterol-dependent processes, focusing first on the postsynaptic side. All subsequent experiments compare control neurons and neurons depleted by 5 min 10 mM MβCD pretreatment—the mildest pretreatment which induces significant changes in both NMDAR and AMPAR eEPSCs.

### Cholesterol modulates the function of NMDARs but not AMPARs

**Figure 2:**
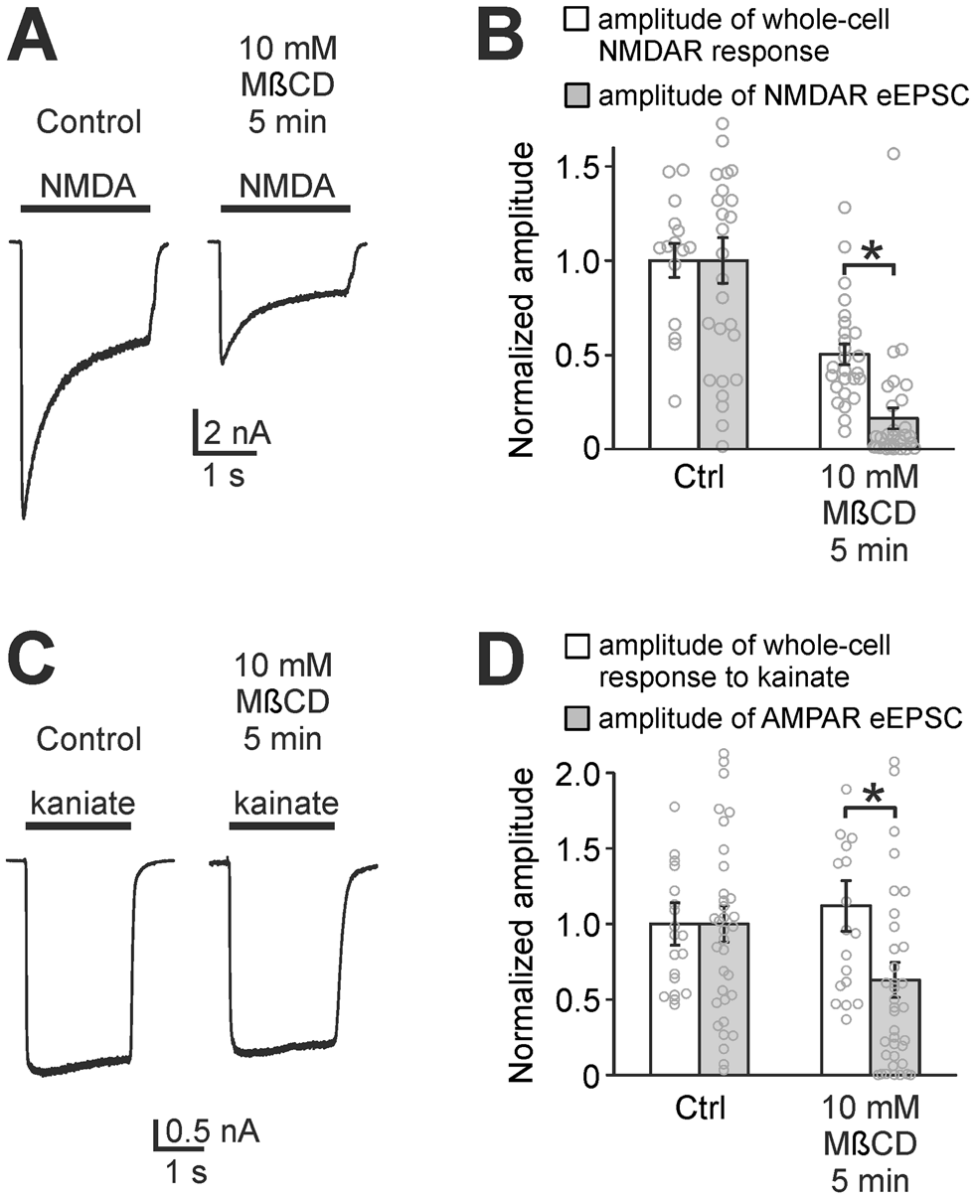
Cholesterol depletion has a bigger impact on eEPSCs compared to whole-cell responses. (**A**) Representative responses of NMDARs induced by whole-cell application of 1 mM NMDA to a control autaptic neuron and a cholesterol-depleted autaptic neuron (10 mM MβCD pretreatment, 5 min). (**B**) Empty bars represent mean amplitudes of whole-cell responses of control and cholesterol-depleted neurons to 1 mM NMDA. The means were normalized (control = 1) and they show the reduction of responsiveness of NMDARs after cholesterol depletion. Filled bars show a significantly greater reduction of amplitudes of NMDAR eEPSCs (same data as shown in Fig. 1B). (**C**) Representative responses of AMPARs (AMPAR stands for AMPA and kainate receptors) induced by whole-cell application of 50 µM kainate to a control neuron and a cholesterol-depleted neuron. (**D**) Normalized amplitudes of whole-cell responses to 50 µM kainate (empty bars) show the unchanged whole-cell responses of AMPARs in control conditions and after cholesterol depletion. Filled bars represent amplitudes of AMPAR eEPSCs (same data as shown in Fig. 1G) which show a significant reduction compared to whole-cell responses. (*p < 0.05, MW test).

To observe whether cholesterol depletion-induced reduction of NMDAR eEPSCs can be explained simply by the decrease in the function of NMDARs^3^, we compared the amplitudes of NMDAR eEPSCs with NMDAR whole-cell responses to fast application of 1 mM NMDA on excitatory autaptic neurons. Cholesterol depletion (5 min, 10 mM MβCD) induced a 50 ± 5% reduction of whole-cell responses to a saturating concentration of 1 mM NMDA (Fig. 2A,B). However, the same depletion induced a significantly larger reduction (84 ± 6%, *p* < 0.001, MW test) of NMDAR eEPSCs (Figs. 1B and 2B).

Similarly, whole-cell responses to the AMPA and kainate receptor agonist kainate were compared with AMPAR eEPSCs (AMPAR stands for AMPA and kainate receptors as defined in the Introduction). Kainate at the concentration of 50 µM activates both AMPA receptors (partial activation) and kainate receptors (almost complete activation)^21^. Figure 2C,D shows that cholesterol depletion did not affect whole-cell responses to kainate. On the contrary, AMPAR eEPSCs were reduced (Figs. 1G and 2D). Altogether, these results show that NMDAR function but not AMPAR function is attenuated by cholesterol depletion. This experiment shows that the reduction of NMDAR and AMPAR eEPSCs cannot be explained solely by cholesterol-driven changes of receptor function but other processes, specific to synapses, are also impaired by cholesterol depletion.

### Cholesterol controls NMDAR open probability

Our next experiment focused on a more detailed description of the cholesterol depletion-induced decrease in NMDAR function. Previous results from our laboratory obtained from cultured cerebellar granule cells showed that cholesterol depletion induced a profound reduction of the NMDAR peak open probability (P_o_). NMDAR single channel current, the affinity for glycine and the affinity for NMDA were not changed upon cholesterol depletion^3^.

P_o_ is defined as the ratio of the number of opened NMDAR channels at the peak of the response to a saturating agonist concentration and the total number of NMDARs. To measure the P_o_ for the synaptic pool of NMDARs, we used the method introduced in^22^. It utilizes the fact that there are two kinds of neurons in autaptic cultures: excitatory neurons that have NMDA receptors both in synaptic and extrasynaptic pools, and inhibitory neurons that do not have excitatory synapses and therefore their NMDARs are only extrasynaptic. By comparing the P_o_ of all receptors in excitatory and inhibitory neurons, we can infer the P_o_ of the synaptic pool of NMDAR^22^. The advantage of this approach is that P_o_ measurement is not skewed by NMDAR dynamic exchange between synaptic and extrasynaptic pools^23^.

**Figure 3:**
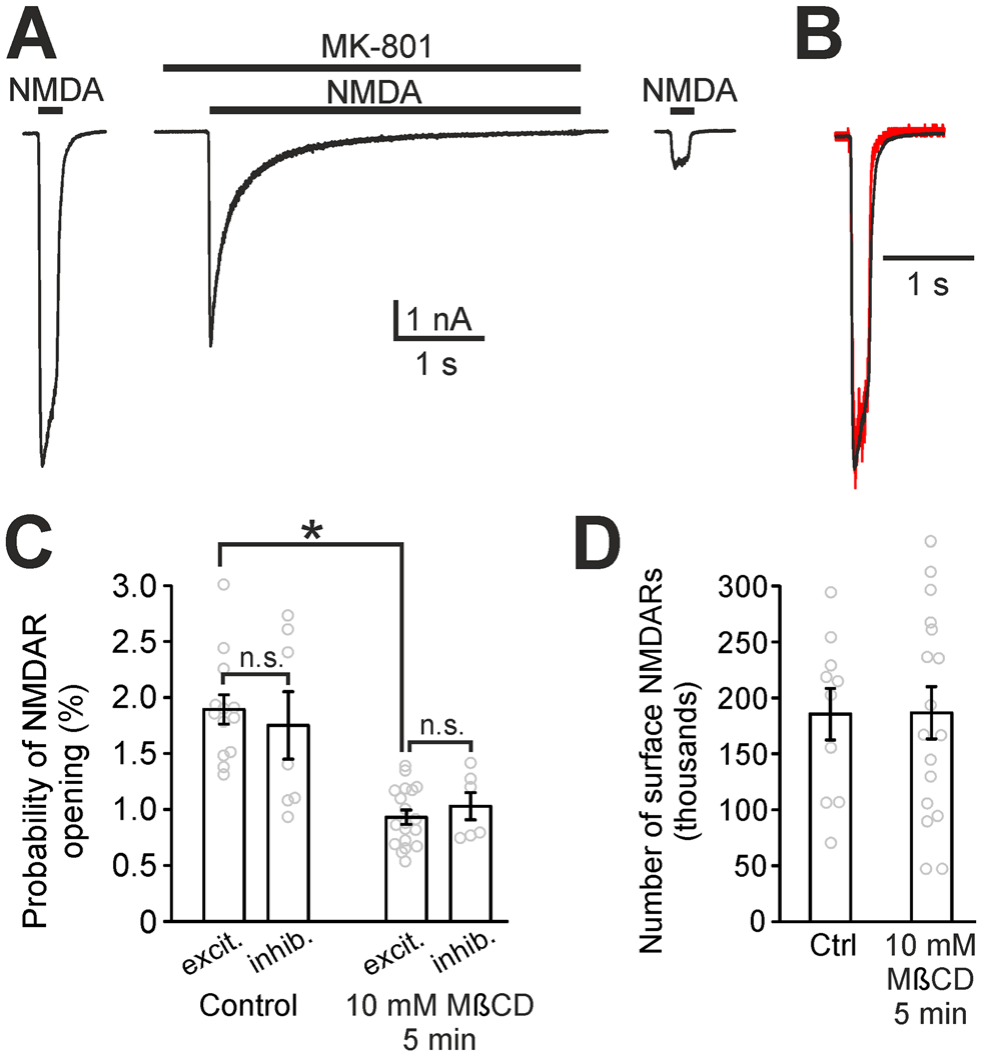
Cholesterol depletion reduces the probability of opening of synaptic NMDARs. (**A**) The three-step protocol we used to measure the P_o_ and the number of surface NMDARs. The principle of the measurement is explained in the Methods. (**B**) A superimposition of normalized NMDAR responses to NMDA before and after MK-801 application (red trace). (**C**) The comparison of P_o_ of NMDARs in control autaptic neurons and in cholesterol-depleted autaptic neurons (10 mM MβCD pretreatment, 5 min). Excitatory neurons (excit.), which contain synaptic and extrasynaptic NMDARs, are compared with inhibitory neurons (inhib.) containing solely extrasynaptic NMDARs. n = 6 to 19 neurons for each bar. (n.s. stands for no significant difference). (**D**) The comparison of the number of surface NMDARs in excitatory neurons assessed by the three-step protocol. n = 10 and 17, respectively. (*p < 0.05, t-test).

We measured P_o_ using a three-step protocol^24,25^ (see the Methods, Fig. 3A,B) and found that in control excitatory neurons, whole-cell P_o_ is 1.89 ± 0.13% (n = 13, Fig. 3C). Cholesterol depletion (5 min 10 mM MβCD) induced a 49% decrease in the P_o_ of excitatory neurons: P_o_ = 0.93 ± 0.06% (n = 19, *p* < 0.0001, t-test). Inhibitory neurons followed the same trend (P_o_ = 1.75 ± 0.30% for control and 1.03 ± 0.12% for depleted neurons) and their P_o_ values do not differ significantly from excitatory neurons either in control conditions or in depleted neurons (Fig. 3C). These measurements suggest that the P_o_ of synaptic and extrasynaptic NMDARs is the same and P_o_ is reduced equally in both pools, by approximately 50%, after cholesterol depletion. Therefore, our findings indicate that one of the crucial roles of naturally occurring cholesterol in the maintenance of synaptic transmission is the regulation of synaptic NMDAR P_o_, potentiating it to appropriate physiological values.

By using the three-step agonist application protocol we could also calculate the number of NMDARs in the plasma membrane (see the Methods). Figure 3D shows that cholesterol depletion did not change the number of NMDARs in the plasma membrane of neurons.

### Cholesterol stabilizes NMDARs in synapses

**Figure 4:**
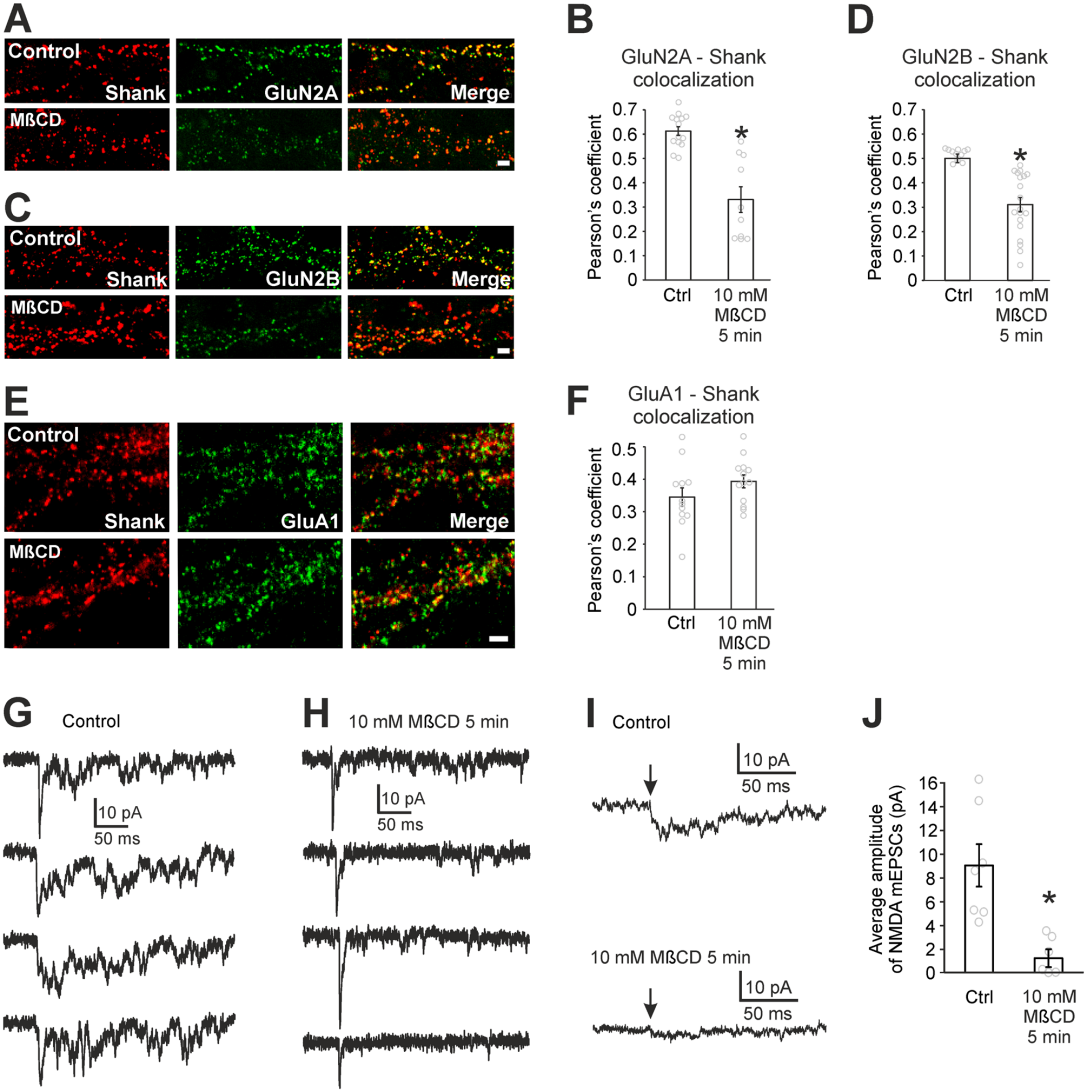
Cholesterol depletion reduces synaptic localization of NMDARs. (**A**,**C**) Colocalization of surface GluN2A (A, green) or GluN2B (C, green) and the postsynaptic marker Shank (red) in control and cholesterol-depleted neurons (10 mM MβCD pretreatment, 5 min). Scale bar 2 µm. (**B**,**D**) Bar graphs showing Pearson's coefficient for the colocalization indicate the reduction of synaptic localization of GluN2A and GluN2B after cholesterol depletion. (**E**) Colocalization of surface GluA1 (green) and the postsynaptic marker Shank (red) in control and cholesterol-depleted neurons (MβCD). Scale bar 2 µm. (**F**) Bar graph showing Pearson's coefficient for the colocalization. (**G**) Examples of typical dual AMPAR-NMDAR mEPSCs in control autaptic neurons having various AMPAR to NMDAR ratio. (**H**) Examples of typical dual AMPAR-NMDAR mEPSCs in 10 mM MβCD-pretreated autaptic neurons. (**I**) Examples of NMDAR mEPSCs obtained from average dual mEPSCs after AMPAR mEPSC subtraction. A control neuron (top trace) and a cholesterol-depleted neuron (bottom trace). The arrows indicate mEPSC onsets. (**J**) The comparison of average amplitude of NMDAR mEPSCs in control neurons and in cholesterol-depleted neurons. (*p < 0.05 relative to control, t-test).

The fact that cholesterol depletion did not change the number of NMDARs in the plasma membrane does not exclude the possibility that cholesterol depletion induces redistribution of NMDARs away from the synapse and into extrasynaptic plasma membrane, which would contribute to the reduction of NMDAR eEPSCs in cholesterol-depleted neurons. Therefore, we used immunocytochemical staining of hippocampal mass cultures to test whether acute cholesterol depletion (5 min, 10 mM MβCD) affects the distribution of surface GluN2A and GluN2B containing NMDARs. Similar immunocytochemical experiment was focused on the surface distribution of GluA1 containing AMPARs. In control cells, surface NMDARs exhibited the characteristic punctate pattern (Fig. 4 A,C) along the dendrites and in the synapses (labelled with Shank, a scaffolding protein in the postsynaptic density^26^). Cholesterol depletion markedly reduced the punctate pattern, making the labelling more diffuse (Fig. 4A,C). Using colocalization analysis (synapses labelled with Shank), we quantified the effect of cholesterol depletion on synaptic localization of surface NMDAR and found a strong reduction of synaptic GluN2A in cholesterol-depleted neurons (Pearson’s coefficient 0.60 ± 0.01 in control compared to 0.33 ± 0.05 in cholesterol-depleted neurons; *p* < 0.0001, t-test, Fig. 4B) and a significant reduction in the synaptic content of GluN2B (Pearson’s coefficient 0.52 ± 0.02 in control compared to 0.31 ± 0.03 in cholesterol-depleted neurons; *p* = 0.0005, t-test, Fig. 4D) without affecting GluA1 containing AMPAR (Pearson’s coefficient 0.34 ± 0.02 in control compared to 0.38 ± 0.02 in cholesterol-depleted neurons; *p* = 0.17, t-test, Fig. 4E,F). In control samples, Pearson’s coefficient for GluA1-Shank colocalization is smaller than that for GluN2A-Shank and GluN2B-Shank. This is probably due to the fact that young synapses contain a smaller number of AMPARs. As development progresses, synapses acquire AMPARs with little change in the NMDAR number^27^. These observations suggest that cholesterol depletion results in a redistribution of surface GluN2A and GluN2B NMDARs from synaptic to extrasynaptic plasma membrane while GluA1 AMPARs do not leave synapses after cholesterol depletion.

To further strengthen microscopy observations, we used electrophysiology to measure whether the amplitudes of NMDAR miniature EPSCs (mEPSCs) are reduced following cholesterol depletion in excitatory autaptic neurons. To get average NMDAR mEPSCs, we measured dual AMPAR-NMDAR mEPSCs and AMPAR mEPSCs. Dual AMPAR-NMDAR mEPSCs in control and cholesterol-depleted neurons show a marked difference in the NMDA component (Fig. 4G,H). For each cell, average NMDAR mEPSC (Fig. 4I) was found by subtracting the average AMPAR mEPSC from the average dual AMPAR-NMDAR mEPSC^28^. Average NMDAR mEPSC amplitude estimated by this method was 9.0 ± 1.8 pA (n = 7) in controls and it diminished by 84% to 1.4 ± 0.6 pA (n = 6) in cholesterol-depleted (5 min, 10 mM MβCD) neurons (*p* = 0.005, t-test, Fig. 4J). Such decrease in NMDAR mEPSC amplitude can be partially explained by the above-described reduction of P_o_ of NMDARs. However, the observed reduction of NMDAR mEPSC amplitude is greater than the observed reduction of P_o_, consistent with the observation that cholesterol depletion reduces synaptic NMDAR content.

### Cholesterol alters lateral diffusion of synaptic NMDARs

**Figure 5:**
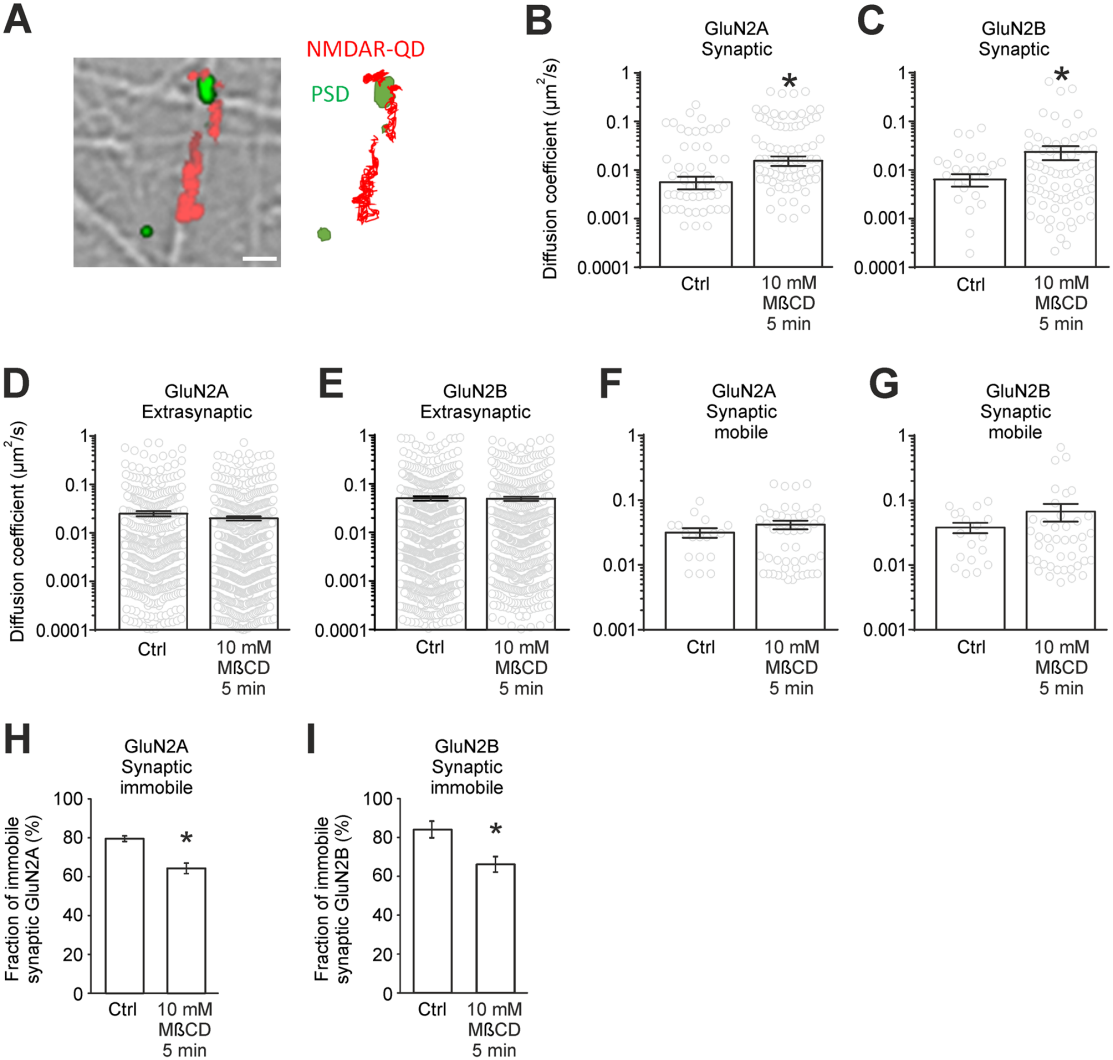
Cholesterol depletion reduces the fraction of synaptic immobile NMDARs. (**A**) Surface NMDARs were detected using a QD-antibody complex directed against extracellular epitopes in GluN2A or GluN2B. Left, representative summed trajectories of NMDAR-QDs (red) acquired over a period of 25 s (20 Hz frame rate) in hippocampal neurons. Scale bar 5 µm. Right, representative examples of NMDAR reconstructed trajectories. (**B**,**C**) Diffusion coefficients for synaptic GluN2A-containing NMDARs and GluN2B-containing NMDARs in control and after cholesterol depletion (10 mM MβCD pretreatment, 5 min). (**D**,**E**) Diffusion coefficients for extrasynaptic GluN2A-containing NMDAR and GluN2B-containing NMDARs in control and after cholesterol depletion. (**F**,**G**) Diffusion coefficients for the mobile fraction of synaptic GluN2A and GluN2B-containing NMDARs in control and after cholesterol depletion. (**H**,**I**) Fraction of synaptic immobile receptors in control and after cholesterol depletion. (*p < 0.05 relative to control).

One possible mechanism underlying the cholesterol depletion-induced redistribution of surface NMDARs may be an alteration of receptor diffusion. Once at the cell surface, NMDARs diffuse within the plasma membrane, a process that continuously supplies receptors to and removes them from synapses. To address this possibility, we analyzed NMDAR surface dynamics in hippocampal mass cultures by using fluorescent single nanoparticle (quantum-dot, QD)-conjugated antibody to track individual GluN2A or GluN2B-containing NMDARs at the surface of live neurons (Fig. 5A). In living neurons, acute cholesterol depletion (5 min 10 mM MβCD) strongly increased the diffusion coefficient of synaptic GluN2A (Fig. 5B; n = 111 and 140 trajectories in control and cholesterol-depleted neurons, respectively, from at least 3 different cultures; *p* = 0.002, MW test) and GluN2B containing NMDARs (Fig. 5C, n = 60 and 124 trajectories in control and cholesterol-depleted neurons, respectively, from at least 3 different cultures; *p* = 0.006, MW test) with no significant changes in the diffusion coefficient of the extrasynaptic fraction of these receptors (Fig. 5D,E; GluN2A n = 612 and 909 trajectories in control and cholesterol-depleted neurons, respectively, from at least 3 different cultures, *p* = 0.07, MW test; GluN2B n = 911 and 661 trajectories in control and cholesterol-depleted neurons, respectively, from at least 3 different cultures, *p* = 0.98, MW test). We then analysed whether the changes in synaptic NMDAR diffusion may reflect changes in the proportion of immobile NMDARs (defined as those with a diffusion coefficient < 0.005 µm^2^/s) and/or in the diffusion properties of the mobile pool. Unexpectedly, when cholesterol is depleted, there is not a significant difference in the diffusion coefficients of mobile synaptic GluN2A-NMDARs (Fig. 5F, n = 18 and 50 trajectories in control and cholesterol-depleted neurons, respectively, from at least 3 different cultures; *p* = 0.10, MW test) or GluN2B-NMDARs (Fig. 5G, n = 18 and 42 trajectories in control and cholesterol-depleted neurons, respectively, from at least 3 different cultures; *p* = 0.36, MW test). However, cholesterol depletion induced a 15% decrease of the fraction of immobile synaptic GluN2A-NMDARs (Fig. 5H, 79.5 ± 1.5% in control and 64.3 ± 2.7% in cholesterol-depleted neurons, n = 70 and 90 trajectories in control and cholesterol-depleted neurons, respectively, from at least 3 different cultures, *p* < 0.001) and an 18% decrease of immobile GluN2B-NMDAR (Fig. 5I, 84.0 ± 4.5% in control *vs.* 66.1 ± 4.2% in cholesterol-depleted neurons, n = 42 and 82 trajectories in control and cholesterol-depleted neurons, respectively, from at least 3 different cultures, *p* < 0.001). Together, colocalization analysis, electrophysiology and single-particle tracking show that the plasma membrane levels of cholesterol control the synaptic abundance of GluN2A and GluN2B-NMDARs. The mechanism of this phenomenon is based on the cholesterol depletion-induced reduction of the fraction of NMDARs that are kept in synapses (immobile receptors).

### Cholesterol modulates presynaptic steps of synaptic transmission

**Figure 6:**
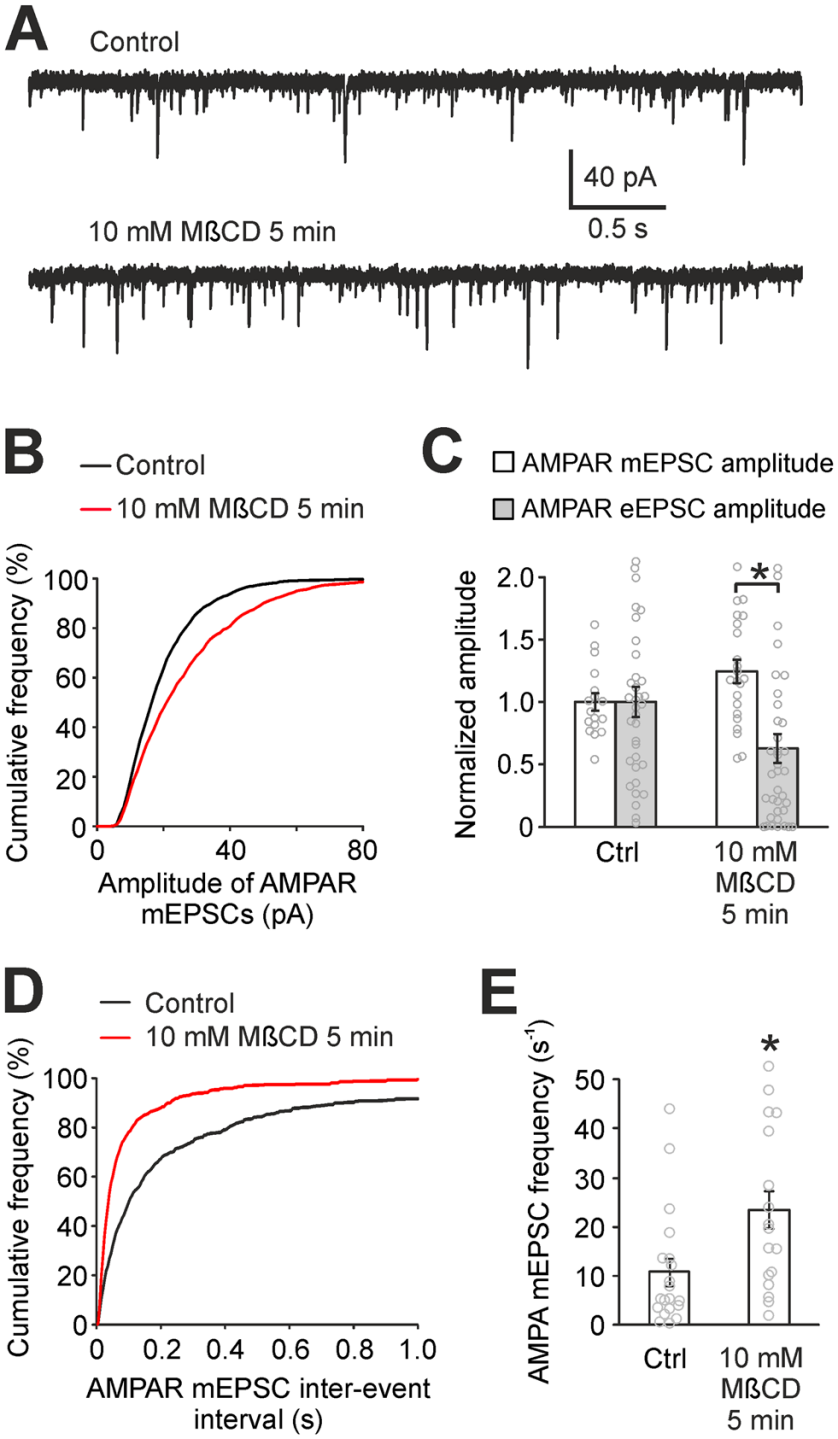
Presynaptic steps of synaptic transmission are impaired by cholesterol depletion. (**A**) Typical recordings of AMPAR mEPSCs in control conditions and after cholesterol depletion (10 mM MβCD pretreatment, 5 min) in autaptic neurons. (**B**) Cumulative histogram of AMPAR mEPSC amplitudes showing positive postsynaptic effect of cholesterol depletion on AMPARs. (**C**) Mean AMPAR mEPSC amplitudes in control conditions and after cholesterol depletion (empty bars). Filled bars represent AMPAR eEPSCs (same data as shown in Fig. 1G). Contrary to AMPAR mEPSCs, AMPAR eEPSCs decrease significantly (*p < 0.05, MW test) after cholesterol depletion, which indicates that glutamate is released at fewer synapses after cholesterol depletion. (**D**) Cumulative histogram of AMPAR mEPSC inter-event intervals in control neurons and in cholesterol-depleted neurons. n = 20 and 18, respectively. (**E**) The comparison of mean AMPAR mEPSC frequencies in control neurons and in cholesterol-depleted neurons (*p < 0.05, MW test).

Next, we turned to AMPAR-mediated synaptic transmission to find whether presynaptic or postsynaptic mechanisms contribute to the reduction of AMPAR eEPSC amplitudes in cholesterol-depleted autaptic neurons. To address this question, we have examined spontaneous glutamate release by recording AMPAR mEPSCs (Fig. 6A–C). The recording was done in the presence of 0.5 µM TTX, 10 µM strychnine, 10 µM bicuculine and 50 µM AP-5. The median amplitude of AMPAR mEPSCs showed a mild increase from 17.0 ± 1.2 pA (n = 17) in controls to 21.1 ± 1.6 pA (n = 22, *p* = 0.04, t-test) in cholesterol-depleted neurons (5 min, 10 mM MβCD). Figure 6C compares the effect of cholesterol depletion on average AMPAR mEPSC (25% increase) with the effect on average AMPAR eEPSC (37% decrease, Fig. 1C). The increase of AMPAR mEPSCs in cholesterol-depleted neurons shows that in the case of AMPARs, postsynaptic function is slightly amplified. Therefore, we infer that cholesterol depletion reduces AMPAR eEPSCs by a presynaptic impairment of evoked synaptic transmission—glutamate is released at fewer synapses after stimulation. Since the presynaptic steps of synaptic transmission are common for both AMPAR and NMDAR eEPSCs, the impairment of presynaptic steps induced by cholesterol depletion is also expected to contribute to the reduction of NMDAR eEPSCs.

### Cholesterol and presynaptic glutamate release

Cholesterol depletion results in fewer presynaptic terminals to release glutamate (Fig. 6C). Therefore, we considered the possibility that cholesterol depletion may modulate presynaptic glutamate release. To assess the effect of cholesterol on glutamate release, we analysed the frequency of AMPAR mEPSCs, paired-pulse ratio of AMPAR eEPSCs, and an effect of ionomycin on mEPSCs in autaptic neurons.

Cholesterol depletion (5 min 10 mM MβCD) significantly increased the frequency of AMPAR mEPSCs which corresponds to a shortening of inter-event intervals (Fig. 6D,E). The frequency increased from 10.7 ± 2.7 s^−1^ (n = 20) in control neurons to 23.4 ± 3.8 s^−1^ (n = 18) in cholesterol-depleted neurons (*p* = 0.005, MW test). It shows that cholesterol depletion facilitates spontaneous glutamate release in synapses.

**Figure 7:**
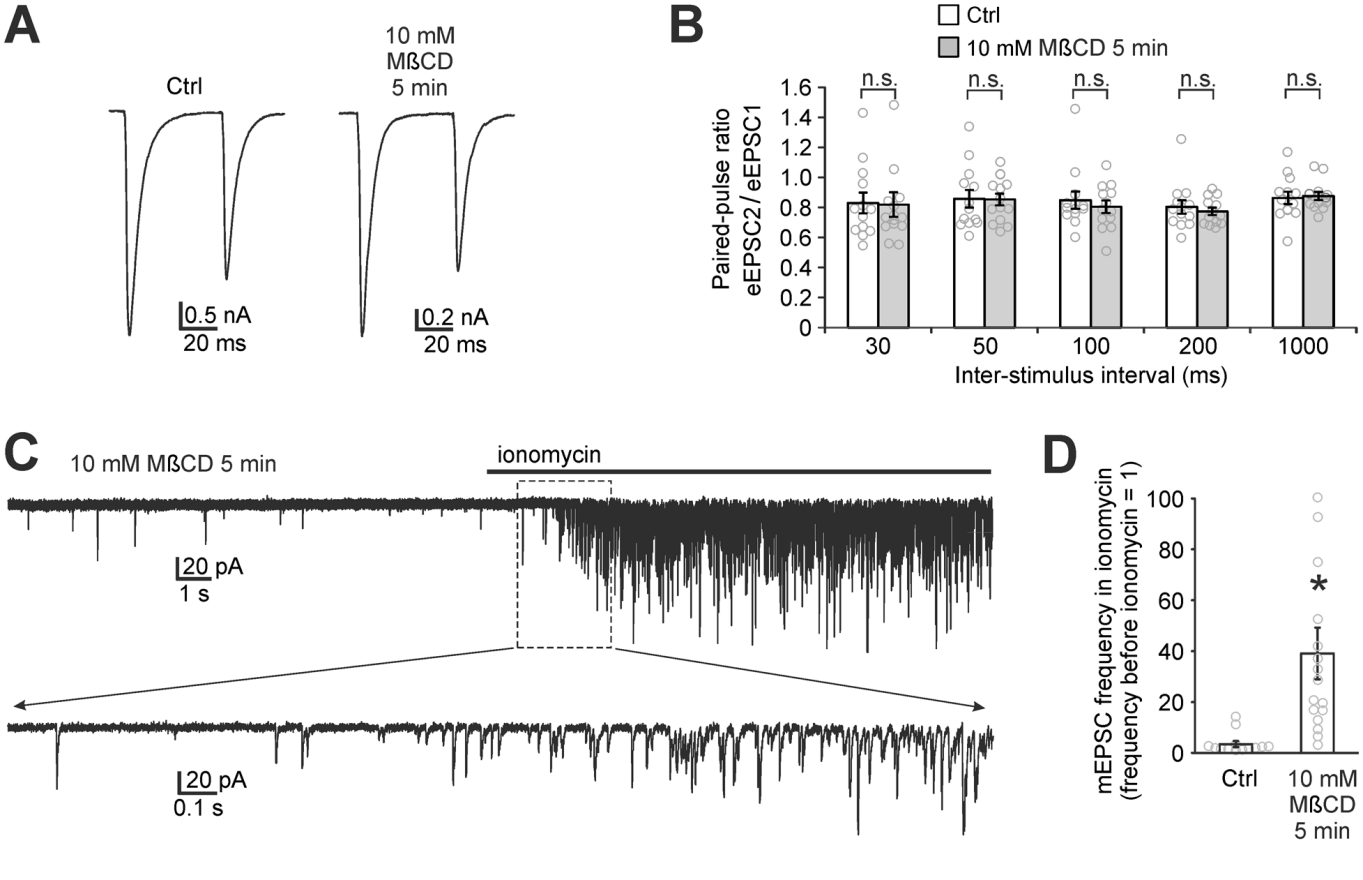
Cholesterol depletion does not reduce the probability of glutamate release. (**A**) Paired-pulse measurement of AMPAR eEPSCs evoked by a pair of stimuli (50 ms inter-stimulus interval) in a control neuron and in a cholesterol-depleted neuron (10 mM MβCD pretreatment, 5 min) in autaptic culture. Action potential signals were removed for clarity. (**B**) Mean paired-pulse ratios (eEPSC2/eEPSC1) in control neurons and in cholesterol-depleted neurons do not differ significantly (n.s.) for any of the inter-stimulus intervals which indicates that the probability of glutamate release is not reduced by cholesterol depletion. (**C**) Typical recording from 10 mM MβCD-pretreated autaptic neuron exposed to application of ionomycin (0.5 µM). Ionomycin-induced entry of calcium into presynapses causes a substantial increase of the frequency of AMPAR mEPSCs. Bottom trace shows the detail of the recording. (**D**) The increase of AMPAR mEPSC frequency induced by ionomycin in control and MβCD-pretreated autaptic neuron. Each data point represents the ratio of the mEPSC frequency after 15 s of ionomycin to the frequency before ionomycin (*p < 0.05 relative to control, MW test).

To test the effect of MβCD pretreatment on the probability of glutamate release, we measured the paired-pulse ratio of AMPAR eEPSCs. The probability of glutamate release can change e.g. as a result of the above-mentioned increase in spontaneous release which might lead to a partial depletion of the available vesicle pool in cholesterol-depleted neurons. The paired-pulse ratio of AMPAR eEPSCs using various inter-stimulus intervals (from 30 ms to 1000 ms) did not show any significant difference between control neurons and 10 mM MβCD-pretreated neurons (t-test p values range from 0.55 to 0.95, Fig. 7A,B). Therefore, paired-pulse measurement does not reveal any change in the probability of evoked glutamate release.

To test whether cholesterol depletion may impair processes occurring in the presynaptic terminal after the arrival of the action potential, we applied calcium ionophore ionomycin on patched autaptic neurons. Ionomycin increases the frequency of mEPSCs by transporting calcium ions into presynapses, which triggers calcium-dependent vesicle release^29^. 0.5 µM ionomycin induced a 39.1 ± 10.1-fold increase of the AMPAR mEPSC frequency in cholesterol-depleted neurons (n = 16, Fig. 7C,D), which is significantly greater compared to a 3.4 ± 1.2-fold increase in control neurons (n = 13); *p* < 0.001, MW test). This result, together with the fact that baseline mEPSC frequency is higher in cholesterol-depleted neurons (Fig. 6D), suggests that cholesterol depletion actually facilitates spontaneous release.

We noted in all our experiments that some cholesterol-depleted neurons (but very few control neurons) have no eEPSCs even though they have AMPAR mEPSCs and they fire action potentials in response to stimulation. In our ionomycin experiment, there were three such neurons, in which MβCD pretreatment caused a complete disappearance of eEPSCs. Interestingly, ionomycin induced a marked increase (3.1, 16.9 and 19.5-fold) of the frequency of AMPAR mEPSCs also in these neurons. This shows that processes occurring in the presynapse following calcium entry and leading to glutamate release are functional after MβCD pretreatment. Therefore, the cause of reduction of AMPAR eEPSCs has to precede calcium entry into the presynaptic terminal or calcium entry itself can be impaired.

Taken together, paired-pulse measurements and the ionomycin experiment suggest that cholesterol depletion causes neither the reduction of the probability of glutamate release nor an impairment of the release process downstream of calcium entry into the presynapse.

### Cholesterol plays a role in action potential propagation

**Figure 8:**
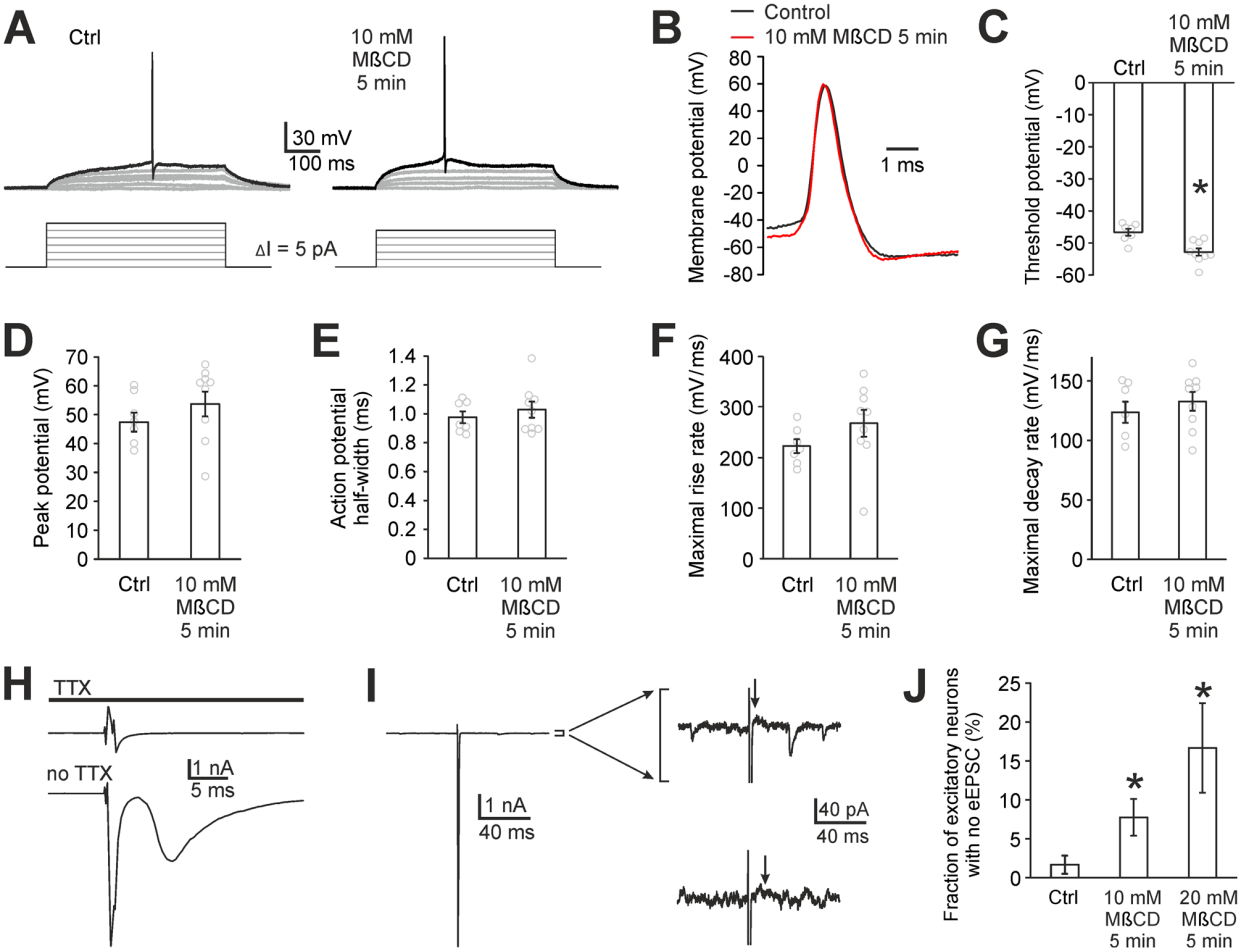
Cholesterol depletion does not disturb the initiation of action potential but likely impairs action potential propagation. (**A**) Typical somatic action potentials evoked in current-clamp mode in autaptic neurons using the step current injection protocol. Bottom schemes show depolarizing current injections increasing by 5 pA each step until the first action potential was evoked. (**B**) An overlay of time courses of somatic action potentials shows their similarity with the exception of the threshold potential. (**C-G**) First action potential in each neuron was analysed for the threshold potential, peak potential, half-width, maximal rise and decay slopes. Control and 10 mM MβCD-pretreated neurons differ significantly only in the threshold potential with MβCD-pretreated neurons being more excitable (*p < 0.05 relative to control, t-test). (**H**) Stimulation carried out in voltage-clamp mode in the presence (top trace) and absence (bottom trace) of TTX shows that the stimulation artefact (top trace) size has similar positive and negative maxima (typically around + 1 nA and -1 nA) while the action potential current amplitude is typically -10 nA (bottom trace, sharp downward-oriented peak). Therefore, a large downward-oriented peak indicates clearly even in the voltage-clamp mode that a patched neuron (e.g. that in panel I) fires an action potential. (**I**) Example recording from a cholesterol-depleted neuron where stimulation evokes an action potential (left trace) but it does not induce any eEPSC. The right top trace is the enlargement of the left one and it was recorded in the presence of AP-5, no glycine. It shows the stimulation artefact (sharp upward peak), the action potential current (downward peak, not complete), random AMPAR mEPSCs and the arrow indicating the time point where typical AMPAR eEPSCs have a maximum. The right bottom trace was recorded in the same neuron in the presence of glycine and CNQX. The baseline is noisy here due to NMDAR activation in overlapping NMDAR mEPSCs. The arrow indicates where typical NMDAR eEPSCs have a maximum. (**J**) Fraction of autaptic neurons which have no eEPSCs. Error bars represent standard deviations of binominal distribution. (*p < 0.05 relative to control).

The above-mentioned experiments suggest that some of the steps of synaptic transmission which precede calcium entry into the presynapse or calcium entry itself are impaired by cholesterol depletion. We observed action potential currents evoked by a depolarizing pulse in all control and cholesterol-depleted neurons (Fig. 8H).

To test the effect of MβCD pretreatment on excitability of autaptic neurons, we measured somatic action potential time courses in current-clamp mode using a step current injection protocol (Fig. 8A–G)^30^. The analysis of action potential properties showed that cholesterol-depleted neurons are slightly more excitable compared to controls: threshold potential was − 46.6 ± 1.1 mV (n = 7) in control neurons and − 52.8 ± 1.1 mV in 10 mM MβCD-pretreated neurons (n = 9, values uncorrected for liquid junction potential, *p* = 0.001, t-test, Fig. 8C). Other parameters describing action potential time course (peak potential, half-width, maximal rise and decay slope) did not change significantly (Fig. 8D–F). This indicates that cholesterol might control the threshold potential for activation of voltage-gated sodium channels. These data suggest that the initiation of the action potential is not disturbed but rather facilitated by cholesterol depletion.

The above-described ionomycin and current-clamp experiments suggest two hypotheses why AMPAR eEPSCs might be reduced in MβCD-pretreated neurons. Either the action potential propagation through cholesterol-depleted axon is disrupted, or the action potential arrives to presynapses but the calcium influx through voltage-gated calcium channels is reduced. However, the reduction of calcium influx typically induces an increase in paired-pulse ratio^31^, which we did not observe. Therefore, we consider the hypothesis of disruption of action potential propagation as the most probable explanation of the AMPAR eEPSC reduction in MβCD-pretreated neurons.

Depending on the location(s) in the branching axon where the propagation is disrupted, the eEPSCs can be reduced to various extents. This hypothesis corresponds well with our experiments. As mentioned above, some cholesterol-depleted neurons have no eEPSCs even though they have AMPAR mEPSCs and they fire action potentials in response to stimulation. Figure 8I shows a recording from one such neuron where AMPAR mEPSCs (single synapse events) are clearly visible while eEPSC is missing completely. As one axon serves all synapses in an autaptic neuron, it is likely that the disruption(s) of action potential propagation occurs in these neurons before the axon reaches the first synapses. Such neurons are able to fire action potentials and release glutamate spontaneously but they fail to show even a single-synapse evoked EPSC (Fig. 8I). Neurons with no EPSCs also appear in control autaptic cultures but their incidence is minimal: 1.7% (2 out of 120). The incidence increased significantly as a result of 10 and 20 mM MβCD pretreatment: 8% (10 out of 129) and 17% (7 out of 42), respectively (Mann–Whitney test *p* = 0.03 and *p* < 0.001, respectively, Fig. 8J).

Together, our results show that the presynaptic function of membrane cholesterol is complex and that cholesterol regulates spontaneous and evoked glutamate release differently. Specifically, our data suggest that naturally occurring cholesterol plays a role in action potential propagation, inhibits spontaneous glutamate release and affects action potential threshold.

## Discussion

**Figure 9:**
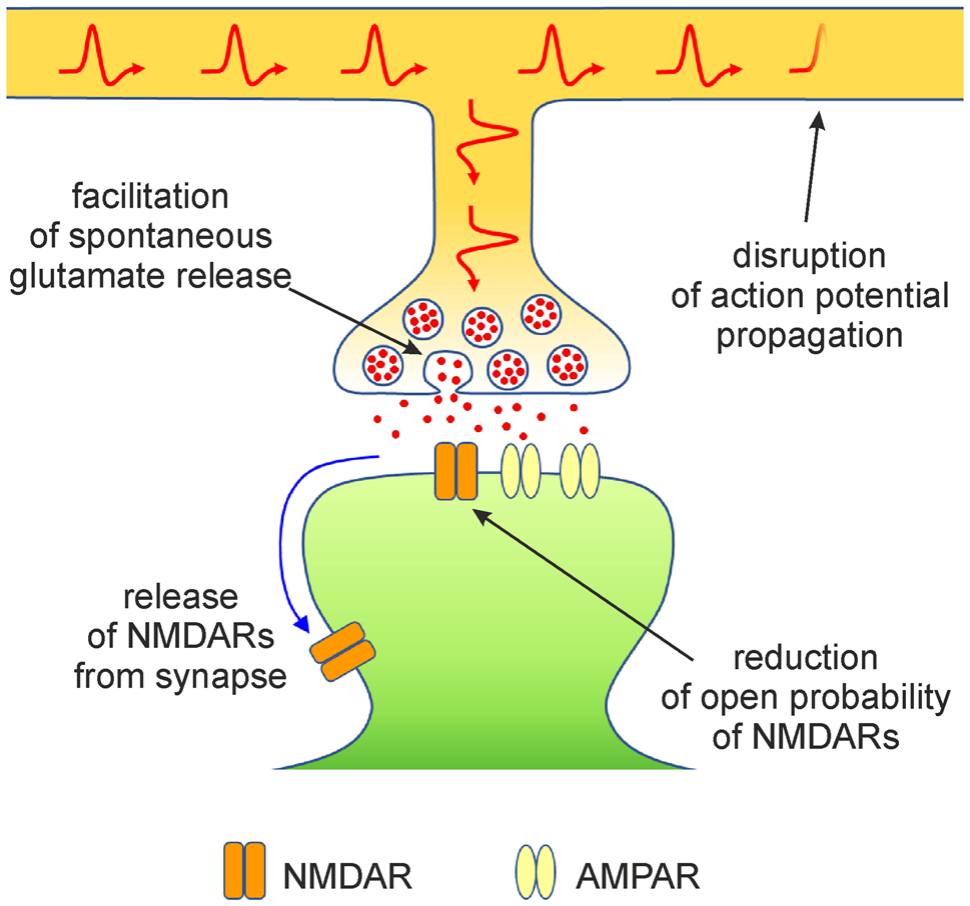
Multiple roles of cholesterol in glutamatergic synaptic transmission. The scheme shows processes affected by acute cholesterol depletion, thus summarizing components of synaptic transmission where endogenous cholesterol plays an active role. Disruption of action potential propagation is shown here as it is the most probable hypothesis explaining AMPAR eEPSC decrease after cholesterol depletion. Cholesterol depletion-induced synaptic delivery of AMPARs is not included in the scheme as this phenomenon is relatively weak (25% increase in synaptic AMPARs in our experimental conditions) compared to other phenomena in the scheme.

In this study, we assessed the role of cholesterol in the physiology of glutamatergic synaptic transmission. We used acute cholesterol depletion of hippocampal cultures by MβCD as a tool to identify synaptic processes where naturally occurring cholesterol plays an active role. We show here that cholesterol depletion induces a significant reduction of amplitudes of both NMDAR and AMPAR eEPSCs. Moreover, cholesterol depletion shortens the deactivation of NMDAR eEPSCs. Our further experiments explored the role of cholesterol in synapses in more detail: the postsynaptic role of cholesterol is to maintain the physiological value of P_o_ of NMDARs and to stabilize NMDARs in the postsynaptic membrane. In contrast, naturally occurring cholesterol appears to moderately downregulate synaptic AMPAR content. Presynaptically, physiological levels of cholesterol appear to be important for action potential propagation along the axon and for attenuation of spontaneous synaptic vesicle release (Fig. 9).

The literature describes several other methods of cholesterol manipulation e.g. using cholesterol oxidase, statins or other inhibitors of cholesterol synthesis (e.g. AY9944)^10,32^. Chronic forms of cholesterol depletion (by statins or other inhibitors) were not used here since they require several days of inhibitor pretreatment^32,33^. Such approach would actually test neuronal adaptation to decreasing cholesterol and would not help achieve the goals of this study.

Our results (Fig. 3) confirm that naturally occurring cholesterol potentiates NMDAR function acting via increasing the P_o_ of NMDARs^3^. The values of P_o_ measured here in control autaptic neurons (~ 2%) correspond to those reported for the same type of culture^22^ but they are lower than the P_o_ values reported elsewhere^34,35^. This discrepancy is probably caused by fact that our measurement of P_o_ requires recording the NMDAR response to a saturating concentration of agonist. It is probable that thanks to the high influx of ions into dendritic spines we are not able to fully control the membrane potential in the spines of long dendrites^36^. Therefore, local membrane potential in the spines is partially depolarized when saturating concentration of agonist is applied. It results in an underestimation of P_o_ and the P_o_ values presented here have to be understood as relative values. In spite of this, our data clearly show the modulatory effect of cholesterol on P_o_ of NMDARs in the autaptic type of neuronal culture.

As for the mechanism of cholesterol effect on NMDAR P_o_, it was reported that enzymatic exchange of cholesterol to 4-cholesten-3-one, associated with no change in plasma membrane fluidity, showed that 4-cholesten-3-one cannot substitute for cholesterol in its potentiating effect on whole-cell NMDAR responses^3^. In contrast, several other sterols (e.g. 24(S)-hydroxycholesterol) potentiate NMDARs^37,38^. Therefore, cholesterol does not modulate NMDARs indirectly by changing the fluidity of the membrane. Since molecules structurally related to cholesterol differ in their effects on NMDARs, cholesterol action at NMDAR is partially specific.

Our immunocytochemical experiments, single-particle tracking, as well as electrophysiology measurements (Figs. 4, 5) showed the importance of cholesterol for NMDAR stability in the postsynaptic membrane. This is in line with a previous observation that cholesterol depletion changes NMDAR distribution from clustered to more diffuse^39^. Here, we focused specifically on synaptic NMDAR clusters and showed that cholesterol depletion induces the release of NMDARs from synapses. The mechanism of this observed release is based on the reduction of the fraction of immobile synaptic NMDARs in cholesterol-depleted neurons.

The regulatory effect of cholesterol on the synaptic pool of AMPARs, which we observed here (depletion-induced increase of AMPAR mEPSC amplitudes, Fig. 6B,C), corresponds to a pathway suggested by Brachet et al*.*^10^. The pathway is triggered by the induction of long-term potentiation, which is followed by intracellular cholesterol decrease and subsequent incorporation of AMPARs containing GluA1 into synapses. Synaptic incorporation of AMPARs measured here and by Brachet^10^ is a relatively minor phenomenon (we show a 25% increase of AMPA mEPSC amplitudes, Fig. 6C), which is perhaps the reason it was not detected in our colocalization experiments (GluA1 and Shank colocalization, Fig. 4F). Similarly, this effect was not reported in other studies assessing AMPAR properties after cholesterol depletion^5,40,41^.

It should be noted that, contrary to our findings, Brachet et al. reported a potentiating effect of cholesterol depletion on NMDAR eEPSCs^10^. This discrepancy probably occurs due to a different method of cholesterol depletion. Brachet used cholesterol oxidase which does not remove cholesterol but converts it to cholest-4-en-3-one. Other metabolites of cholesterol (e.g. 24-(S)-hydroxycholesterol^38^) have a potentiating effect on NMDARs. Therefore, it is probable that the combined effect of cholesterol depletion and cholest-4-en-3-one synthesis has a different effect on NMDAR eEPSCs than MβCD-based cholesterol depletion.

As for presynaptic steps of synaptic transmission (Figs. 6, 7), we show that cholesterol depletion induces a decrease of AMPAR eEPSC amplitudes and a small increase of AMPAR mEPSC amplitudes. This suggests that glutamate is released at fewer synapses in cholesterol-depleted neurons^40^. This can be due to several reasons, e.g. due to a change in glutamate release probability. The literature provides ambiguous results regarding the role of cholesterol in glutamate release. Studies by Dason et al*.* emphasize the importance of cholesterol in membranes of synaptic vesicles for endocytosis and exocytosis of synaptic vesicles while plasma membrane cholesterol manipulation had no effect on endocytosis and exocytosis^42,43^. Wasser et al*.* suggested that cholesterol depletion facilitates spontaneous release and vesicle recycling while it attenuates evoked release and recycling^41^. A biophysical study on cholesterol-phospholipid liposomes predicted an essential role of cholesterol in the relaxation of bending energy of extremely curved membranes during the fusion of vesicles with the plasma membrane^44^. On the contrary, measurements of the frequency of mEPSCs and the paired-pulse ratio presented here and by others indicate that plasma membrane cholesterol depletion has either no or rather an enhancing effect on synaptic vesicle release^40,41,45^, which is also in accordance with Dason et al. Therefore, we suggest that the reduction of the number of synapses releasing glutamate in cholesterol-depleted neurons is caused by the disruption of some process preceding glutamate release.

The increase of spontaneous and ionomycin-induced vesicle release in MβCD-pretreated neurons seems contradictory to the unchanged paired-pulse ratio after MβCD. It has been shown that the mechanisms of spontaneous and evoked glutamate release do not overlap completely^46^. This opens a possibility for a differential modulation of spontaneous and evoked vesicle release by cholesterol. For instance, the pool of spontaneously released vesicles was shown to be partially distinct from the pool of vesicles released upon stimulation^46^. Wasser et al. published a study describing differences in cholesterol modulation of the recycling of vesicles in spontaneously released pool and in the pool of vesicles released upon stimulation^41^. Her observation is an example of a mechanism of how spontaneous release might be upregulated without affecting evoked release.

We tested the effect of ionomycin-induced calcium entry into presynaptic terminals on the frequency of mEPSCs. Ionomycin induced a significantly higher increase of the frequency of mEPSCs in MβCD-pretreated neurons than in controls (Fig. 7D). This can be caused e.g. by a higher calcium transport efficiency of ionomycin in cholesterol-depleted membrane. However, qualitative result of this measurement is more important: ionomycin induced a marked increase of the frequency in those MβCD-pretreated neurons where no eEPSC was observed. This proves that the presynaptic mechanisms downstream of calcium entry are functional, not impaired by MβCD pretreatment. The cause of the reduction of AMPAR eEPSCs in MβCD-pretreated neurons therefore has to precede calcium entry into presynapses, or calcium entry itself can be impaired. However, the later possibility is typically associated with an increase of paired-pulse ratio^31^, which we did not observe.

Vesicle maturation and priming belong to events preceding calcium entry but their downregulation or blockade in MβCD-pretreated neurons is not probable, as neurons producing no eEPSCs (i.e. theoretically fully blocked) show a substantial increase of mEPSC frequency in ionomycin thus showing that they are actually not fully blocked. Theoretically, the blockade of vesicle maturation and priming after MβCD pretreatment could be limited to some neuronal subpopulation but the fact that the fraction of neurons with no eEPSCs increases with an increase of MβCD concentration (Fig. 8J) makes this unlikely.

As regards presynaptic events preceding calcium entry which occur in relation with action potential, we did not observe any weakening or impairment of action potential triggering in our current-clamp measurements. Therefore, we consider the hypothesis of action potential propagation failure as the most probable cause of AMPAR eEPSC reduction.

Action potential propagation failure was observed in MβCD-treated crayfish motoneuron axons^45^, here we show evidence for it for the first time in mammalian neurons. Depending on the locations in the branching axon where the propagation is disrupted, the eEPSCs are reduced to various extents. The hypothesis of action potential propagation failure is in accordance with the paired-pulse measurements and it explains our observation that some cholesterol-depleted autaptic neurons have mEPSCs, fire action potentials but do not produce even a single-synapse eEPSCs (Fig. 8I,J). Since one axon serves all synapses in an autaptic neuron, the disruption of action potential propagation before reaching the first synapses results in the complete loss of eEPSCs. Interestingly, it was reported that warming cortical slices (42 °C), which makes membranes more liquid, similarly to cholesterol depletion, also results in action potential propagation failure^47^. The search for the mechanism of the disruption of action potential propagation in cholesterol-depleted axons exceeds the scope of this study. It may be related to a change in the function of ion channels involved in action potential propagation, a disturbance in axonal ion homeostasis, a change in axonal electroinsulating properties or the axon can become mechanically fragile and leaky.

Our study describes the role of cholesterol in glutamatergic synaptic transmission but its relevance extends to neurotoxicity and diseases associated with impaired cholesterol metabolism. Several publications showed unanimously that cholesterol depletion of cultured neurons (both by statins or cyclodextrins) has a significant neuroprotective effect in NMDA-induced excitotoxicity^32,33,48^. The results presented here show a significant diminution of NMDAR responses following cholesterol depletion which very likely contributes to cholesterol depletion-induced neuroprotection. Correspondingly, some (but not all) studies suggest that statin users have a better outcome in stroke and that statins may decrease the incidence of dementia (reviewed e.g. in^49,50^).

As regards diseases associated with impaired cholesterol metabolism, Niemann-Pick type C disease is a fatal metabolic storage disorder characterized by the accumulation of cholesterol in lysosomes. This is especially severe in neurons and other lipid-dense regions of the central nervous system and it leads to neurodegeneration^51^. Interestingly, administration of 2-hydroxypropyl-β-cyclodextrin reduced neurodegeneration and increased lifespan in animal models of the disease^52^. Clinical trials using intrathecal administration of 2-hydroxypropyl-β-cyclodextrin in Niemann-Pick type C patients brought health benefits but this therapy was linked to a loss of cochlear outer hair cells and hearing^17,53^. The effect of MβCD and cholesterol on synaptic transmission described here contributes to a better understanding of the processes occurring in Niemann-Pick type C disease and in its cyclodextrin-based therapy.

Reduced cholesterol levels occur in Smith-Lemli-Opitz syndrome which is caused by the decreased function of 7-dehydrocholesterol reductase, an enzyme catalyzing the last step of cholesterol synthesis^54^. Out of many symptoms associated with this syndrome, numerous patients present with autistic behavioural characteristics^55^. Interestingly, autism can be associated with NMDAR mutations which decrease NMDAR function^56^. It is possible that the reduction of the NMDAR component of glutamatergic synaptic transmission described here plays a role in the neurodevelopmental deficit associated with Smith-Lemli-Opitz syndrome.

Cholesterol is a major component of mammalian cell membranes. Changes in brain cholesterol levels have been associated with prenatal and postnatal brain development, aging, cognitive decline and neurodegenerative diseases. Therefore, our findings concerning the role of cholesterol in synaptic transmission afford a deeper insight into the physiology of a healthy brain and help to understand the role of cholesterol in brain dysfunctions at the synaptic level.

## Methods

### Cell cultures

Rat autaptic cultures were used for electrophysiology recordings. The cultures were prepared as described in^57^. Briefly, dissociated neonatal male Wistar rat cortex cells from decapitated animals were cultured in astrocyte growth medium (D-MEM with GlutaMax, 10% foetal calf serum (all from Gibco)). After 8 days in vitro, glutamate (100 µM, 5 min) was added to induce excitotoxic death of neurons. On day in vitro 12, glial cells were trypsinized and seeded (5000 cells/cm^2^) onto coverslips which were covered with small (0.3 mm) collagen/poly-D-lysine islands. On day in vitro 14, glial cells grew into confluent micro-islands. Subsequently, dissociated neonatal male Wistar rat hippocampal neurons were seeded at a very low density (300 cells/cm^2^) onto the micro-islands and cultured in neuronal medium (Neurobasal A, 1% GlutaMax and 2% B-27 supplement (all from Gibco)).

Rat hippocampal mass cultures were used for immunocytochemistry and single-particle tracking. The cultures were prepared as previously described^58^. Briefly, hippocampi from embryonic day 18 Wistar rats of either sex were dissected and the cells were dissociated by enzymatic digestion with trypsin for 15 min and mechanical dissociation. Cells were then plated at a density of 500,000 per 35-mm dish or 50,000 onto 22-mm glass coverslips coated with poly-l-lysine (Sigma-Aldrich). The culture medium was composed of Neurobasal medium (Gibco) supplemented with horse serum (10%), B27 (Gibco) and 2 mM glutamine.

### Changing the cholesterol content in plasma membranes

Cholesterol manipulation was carried out prior to electrophysiology measurements, immunocytochemistry staining or single-particle tracking measurements. No cholesterol manipulation was done during the staining or measurements.

Cholesterol depletion of cultured neurons was performed by bathing coverslips in 5, 10 or 20 mM MβCD (Aldrich, average molecular weight 1310 g/mol) for 5 min at 37 °C. MβCD was dissolved in magnesium-containing extracellular solution (Mg-ECS), which contained (in mM) 160 NaCl, 2.5 KCl, 10 HEPES, 10 glucose, 2 CaCl_2_, and 1 MgCl_2_, pH 7.3.

Cholesterol repletion was performed after cholesterol depletion by bathing cultured neurons for 10 min in Mg-ECS enriched with 3.4/20 mM cholesterol/MβCD complex (Sigma-Aldrich) at 37 °C. Cholesterol/MβCD complex, also called water-soluble cholesterol, contained 4.8% cholesterol (w/w) and 95.2% MβCD (w/w).

Control cultures were incubated at 37 °C in Mg-ECS supplemented with 5, 10 or 20 mM sucrose to mimic the osmolarity of MβCD or cholesterol/MβCD. Manipulation of cellular cholesterol content was terminated by washing the cells in Mg-ECS.

### Electrophysiology

Hippocampal autaptic cultures were used for electrophysiology measurements 12 to 14 days after seeding neurons on glial micro-islands. Patch-clamp technique in the voltage-clamp mode was used to record AMPAR-mediated evoked excitatory postsynaptic currents (eEPSCs), NMDAR-mediated eEPSCs and whole-cell responses evoked by NMDA or kainate application. Recordings were made at room temperature (22–24 °C), 5 to 20 min after cholesterol depletion or repletion. We showed that NMDAR responses in neurons are only mildly temperature-sensitive^59^ so doing these experiments at room temperature does not reduce their physiological relevance.

Patch-clamp pipettes (4–6 MΩ resistance) were pulled from borosilicate glass (BioMedical Instruments) and filled with intracellular solution containing (in mM) 125 potassium gluconate, 15 KCl, 5 EGTA, 10 HEPES, 0.5 CaCl_2_, 2 ATP-Mg^2+^ salt and 0.3 GTP-Na^+^ salt, pH 7.2.

Both NMDAR and AMPAR eEPSCs as well as whole-cell responses to agonist application were measured in magnesium-free extracellular solution (Mg-free ECS) containing (in mM) 160 NaCl, 2.5 KCl, 10 HEPES, 10 glucose and 2 CaCl_2_, pH 7.3. Either 6-cyano-7-nitroquinoxaline-2,3-dione (CNQX) or (2R)-amino-5-phosphonopentanoate (AP-5) (both from Tocris) was applied to pharmacologically isolate NMDAR or AMPAR responses, respectively. Application of agonists and inhibitors was done through a set of parallel tubes moved by a stepper motor. For the measurements of eEPSCs, slow perfusion rate was used (solution exchange around the cell occurred in ~ 1 s). For the whole-cell responses to agonists (NMDA or kainate (both from Sigma-Aldrich)), fast solution application was used, with the solution exchange time of around 30 ms^25^ and with tetrodotoxin added to application solutions. Glycine (10 µM) was applied 3 s before and during the activation of NMDAR. Recordings were made using Axopatch 200B amplifier (Molecular Devices) at a holding potential of − 60 mV. eEPSCs were measured after triggering an action potential by a brief depolarization (1 ms to 0 mV) applied to the pipette electrode. Series resistance (< 10 MΩ) was compensated to 90%. Receptor responses were low-pass filtered (2 kHz eight-pole Bessel filter) and digitally sampled at 10 kHz.

To measure the time course of action potentials, neurons in the current-clamp mode were biased with current injection to set the membrane potential to − 70 mV. Subsequently, a step current injection protocol was used: 500 ms depolarizing current pulses with incrementing amplitudes (0 pA in the first sweep and incrementing by 5 pA each sweep) were applied and the first evoked action potential was analysed.

Excitatory neurons were identified in voltage-clamp mode by AMPAR miniature EPSCs (mEPSCs). Other neurons were not included in the data analysis unless stated otherwise. Data acquisition and analysis were performed using pClamp 10.1 software (Molecular Devices).

### Measurement of the peak open probability of NMDARs

Peak open probability of NMDARs (P_o_) was measured using a three-step protocol established by^24^ and explained in detail in^22,25^. Briefly, we recorded three whole-cell responses to 1 mM NMDA (Fig. 3A) where the second application of NMDA was done in the presence of 10 µM ( +)d-methyl-l0,11-dihydro-5H-dibenzo[a,d]cyclohepten-5,10-imine (MK-801) (Sigma). MK-801 is an almost permanent blocker of NMDARs with the blocking rate constant k_MK_ = 25 µM^−1^ s^−1^ in hippocampal neurons at − 60 mV^24^. In the presence of 10 µM MK-801, an average NMDAR opens cumulatively for

1 $${\text{t}}_{\mathrm{b}} = 1/( {\text{k}}_{\mathrm{MK}} [{\text{MK-801}}]) = 4\,{\text{ms}}$$

before it is blocked. Before an average NMDAR is blocked, it conducts a mean charge:

2 $${\text{q}} = {\text{t}}_{{\mathrm{b}}} {\text{i}},$$

where i is the single channel current. In 2 mM extracellular Ca^2+^ at − 60 mV, i = 2.7 pA^60^. The number of NMDARs blocked during NMDA and MK-801 co-application is:

3 $${\text{N}}_{{\mathrm{b}}} = {\text{Q}}/{\text{q}},$$

where Q is the charge transfer during NMDA and MK-801 co-application. The fraction of NMDARs blocked during NMDA and MK-801 co-application is N_b_/N, where N is the total number of NMDAR in the plasma membrane. This fraction is also equal to (A_1_-A_2_)/A_1_ where A_1_ and A_2_ are the amplitudes of responses to the first and third NMDA applications (Fig. 3A), respectively:

4 $${\text{N}}_{{\mathrm{b}}} /{\text{N}} =({\text{A}}_{1} - {\text{A}}_{2} )/{\text{A}}_{1}.$$

Substituting the Eqs. (3), (2) and (1) to Eq. (4) allows us to calculate the number of surface NMDARs: N = QA_1_k_MK_[MK-801]/(i(A_1_-A_2_)). Since the number of NMDARs opened at the peak of the first response (N_o_) is N_o_ = A_1_/i, we can derive that P_o_ = N_o_/N = (A_1_-A_2_)/(Qk_MK_[MK-801]). We present values of peak open probability in per cents.

### Measurement of NMDAR miniature EPSCs

To obtain NMDAR mEPSCs, dual AMPAR-NMDAR mEPSCs were measured in the presence of 0.5 µM TTX, 10 µM bicuculine, 10 µM strychnine (all from Tocris) and 10 µM glycine (Sigma). Subsequently, 50 µM AP-5 was added and glycine was washed to measure AMPAR mEPSCs from the same cell. To get average NMDAR mEPSC, AMPAR mEPSCs from one cell were aligned, averaged and subtracted from the aligned (AMPA peak was used to align) and averaged dual AMPAR-NMDAR mEPSCs from the same cell^28^. The amplitude of average NMDAR mEPSC was found as the mean of the data in the interval 7 to11 ms after the onset. Different intracellular solution was used in this experiment to minimize baseline noise (in mM): 125 gluconic acid, 15 CsCl, 5 EGTA, 10 Hepes, 3 MgCl_2_, 0.5 CaCl_2_ and 2 ATP-Mg salt (pH-adjusted to 7.2 with CsOH).

### Immunocytochemistry

Neurons were stained in non-permeabilized conditions with primary antibodies against extracellular epitopes of GluN2A, GluN2B or GluA1 (ACG-002 1:500, Alomone Labs^61,62^, ACG-003 1:500, Alomone Labs^61,63^, and PC246 1:1000, MERCK^64^) and then they were depleted of cholesterol. Cells were washed twice, fixed and permeabilized (methanol, − 20 °C, 5 min). After 30 min in blocking solution (10% Horse Serum, PBS), neurons were incubated with Shank antibody (N23B/49 1:1000, MERCK^65,66^) for 1 h. Cells were washed twice and incubated with secondary antibodies (Alexa 555 or Alexa 488-conjugated, 1:1000, A-31572, A-21206 Thermo Fisher) to detect GluN2A/B, GluA1 and Shank (30 min, RT) prior to visualization by confocal fluorescence microscopy.

### Single-particle tracking

For endogenous GluN2A or 2B quantum dot (QD) fluorescence particle tracking, hippocampal neurons were incubated with an antibody against the N-terminal extracellular domain of GluN2A (ACG-002 1:500, Alomone Labs) or GluN2B subunits (ACG-003 1:500, Alomone Labs) for 10 min. Neurons were then washed and incubated for 5 min with QD 655 anti-rabbit IgG (Q-11421MP, Invitrogen^67,68^). Non-specific binding was prevented by adding casein (Vector Laboratories) to the QD 15 min before use. Single-particle tracking was done at 37 °C on an inverted fluorescence microscope Leica DMI600s equipped with a mercury lamp for excitation, appropriate excitation/emission filters and a CCD camera (iXon, Andor) for image acquisition. Images were acquired with an interval of 50 ms and up to 500 consecutive frames. Single-particle tracking was carried out for up to 20 min after the staining and MβCD treatment. QD recording sessions were processed with MATLAB software^69^. The two-dimensional trajectories of single molecules in the plane of focus were constructed by correlation analysis between consecutive images using a Vogel algorithm. The instantaneous diffusion coefficient, D, was calculated for each trajectory, from linear fits of the first 4 points^5^ of the mean square displacement (MSD) vs. time function using MSD(t) =  < r^2^ > (t) = 4Dt. QD-based trajectories were considered synaptic if they colocalized with dendritic clusters (MitoTracker™ Green FM^62,70^) for at least five consecutive frames. Diffusion is a temperature-sensitive process, therefore single-particle tracking experiments were always done with samples at 37 °C in a temperature-controlled chamber to get physiologically relevant data. Importantly, maintaining standard experimental conditions allow us to compare our results with the results obtained by other groups in the field.

### Colocalization analysis

Colocalization analysis was performed as reported before^58^. Briefly, confocal images were analysed using ImageJ software (NIH). The degree of colocalization between immunolabels was assessed by calculating the Pearson's correlation coefficient in the ROI using the JaCoP plugin^71^. The Pearson's correlation coefficient was calculated for the original data and for a large set (500) of images randomized with a grain size determined by the point spread function of the microscope objective. If the Pearson's correlation coefficient of the original image was not greater than 95% of the randomized images, then the samples were not used. Cells displaying saturated or low, near-threshold signals were also discarded. Automated thresholding was used to avoid user bias in setting analysis parameters. Bar graphs show the mean correlation coefficient ± standard error of measurement.

### Statistical analysis

All datasets were tested for normality using Kolmogorov–Smirnov test (SigmaPlot software). Unpaired two-tailed Student's t-test was used to compare datasets with normal distribution. Datasets with non-normal distribution were compared using Mann–Whitney (MW) test (SigmaPlot software). The results are presented as mean ± standard error of the mean with n indicating the number of cells unless stated otherwise. Probability value of *p* = 0.05 was used to decide on statistical significance.

### Ethical approval

All animal experiments were approved by the Animal Care and Use Committee of the Institute of Physiology of the Czech Academy of Sciences and conducted in accordance with the European Union directive 2010/63/EU.

## Data Availability

All data generated or analysed during this study are included in this published article.
